# A Case of Pembrolizumab-Induced Diabetic Ketoacidosis and Hyperthyroidism in a Patient With Recurrent Esophageal Adenocarcinoma

**DOI:** 10.7759/cureus.35276

**Published:** 2023-02-21

**Authors:** Jonathan Salangsang, Surendra Sapkota, Sanjeev Kharel, Prakash Gupta, Abhishek Kalla

**Affiliations:** 1 Internal Medicine, Ascension Saint Agnes Hospital, Baltimore, USA; 2 Internal Medicine, Tribhuvan University Institute of Medicine, Kathmandu, NPL; 3 Internal Medicine, Virgen Milagrosa University Foundation College of Medicine, Pangasinan, PHL; 4 Hematology and Oncology, Ascension Saint Agnes Hospital, Baltimore, USA

**Keywords:** immune-related adverse events, recurrent esophageal adenocarcinoma, hyperthyroidism, diabetic ketoacidosis (dka), pembrolizumab

## Abstract

Immune checkpoint inhibitors (ICI) such as program cell death protein 1 (PD-1) inhibitors are widely used for the treatment of patients with recurrent, locally advanced or metastatic, gastric or gastroesophageal (GE) junction adenocarcinoma. Immune-related adverse events (irAE) such as endocrinopathies have been reported after patients received ICI. We report a case of pembrolizumab-induced hyperthyroidism and type 1 diabetes mellitus (DM1) presenting with diabetic ketoacidosis (DKA). A 53-year-old African American male with no history of diabetes or hyperthyroidism was treated with two cycles of pembrolizumab for recurrent GE junction adenocarcinoma after which he was admitted with hyperthyroidism (thyroid stimulating hormone [TSH] 0.070mIU/L, free thyroxine 1.85mIU/L) and DKA (pH 7.06, glucose 583 mg/dL, beta-hydroxybutyrate 8.63 mmol/L, anion gap 27 meq/L). The patient was treated with intravenous insulin and aggressively hydrated. Given the lack of other precipitating factors for the two endocrinopathies, it was determined that the most likely etiology was recent treatment with pembrolizumab (a PD-1 inhibitor). In our case, pembrolizumab monotherapy developed two irAE (hyperthyroidism and DKA), which is unique as most combined immunotherapy regimens are associated with the development of multiple endocrinopathies. Our case emphasizes the importance of baseline monitoring of thyroid function and blood glucose prior to the start of ICI to monitor and evaluate patients with immune-related adverse events, including endocrinopathies.

## Introduction

Gastroesophageal (GE) junction and gastric adenocarcinomas can be treated with immune checkpoint inhibitors (ICI), which specifically target the cytotoxic T lymphocyte antigen 4 (CTLA-4) and program cell death protein 1 (PD-1) and its receptor. Approximately 40% of GE carcinomas were found to express PD-1 [1]. PD-1 inhibitors have been approved for the treatment of a number of malignant solid tumors that are recurrent, resistant, and metastatic. The PD-1 inhibitor pembrolizumab, which can be used to treat patients with recurrent, locally progressed, or metastatic gastric or gastroesophageal junction adenocarcinoma, was approved by the US Food and Drug Administration (FDA) in 2017 [2].

PD-1 inhibitors, such as pembrolizumab, have shown excellent success in the treatment of cancer, but there has also been an increase in immune-related adverse events (irAE) [2,3]. Thyroid dysfunction and colitis are two of the most common severe side effects associated with ICI. There have also been reports of minor side effects, including fatigue, pruritus, rash, and fever [2,3]. Rarely have people taking pembrolizumab reported having developed type 1 diabetes mellitus (DM1) or even had diabetic ketoacidosis (DKA) [3,4]. Pembrolizumab-induced DKA was reported in only 0.1% of patients participating in clinical trials [5].

In addition, very few examples of multiple endocrinopathies have been reported. In this case report, we describe a patient who developed DM1 that resulted in DKA along with hyperthyroidism after pembrolizumab treatment for recurrent GE junction cancer.

## Case presentation

A 53-year-old African American male with a history of recurrent esophageal carcinoma status post two cycles of pembrolizumab and no history of diabetes mellitus or thyroid disease presented to the emergency department with fatigue for two days. Fatigue was associated with shortness of breath, dry mouth, polyuria, and polydipsia. He said that he had not had any headaches, vision changes, night sweats, fevers, chills, hematemesis, hemoptysis, chest pain, diarrhea, constipation, or paresthesia.

Approximately two years before this admission, the patient was found to have moderately differentiated GE junction adenocarcinoma after an esophagogastroduodenoscopy (EGD) revealed a severe, malignant appearing stenosis 40 cm from the incisors. Positron emission tomography (PET) revealed a localized disease at the GE junction with no metabolically active or pathologically enlarged lymph nodes in the mediastinal or gastrohepatic region. He received three cycles of neoadjuvant carboplatin/paclitaxel and underwent Ivor Lewis esophagectomy with the resulting pathology report indicating ypT3 ypN0 disease. A one-year follow-up EGD revealed several abnormally presenting mucosal lesions with evidence of ulceration and inflammation at the GE junction. The biopsy revealed moderately differentiated recurrent invasive adenocarcinoma. Computed tomography (CT) chest, abdomen, and pelvis (C/A/P) revealed post-esophagectomy and gastric pull-through changes, but no evidence of metastatic disease. The patient was started on capecitabine/oxaliplatin chemotherapy. PET/CT after three cycles of capecitabine/oxaliplatin did not reveal evidence of metabolic activity. Chemotherapy was held after nine cycles as the patient had significant fatigue and nausea. EGD revealed a persistent malignant nodule in the distal esophagus. It was decided to start pembrolizumab for the patient as tumor cells were positive for Programmed death-ligand 1 (PD-L1). He received two doses after which he was admitted to our hospital with acute illness, described below.

On admission, he was afebrile, had a respiratory rate of 32 per minute, a heart rate of 113 per minute, and an oxygen saturation of 98% on room air. Physical examination revealed drowsiness, dry oral mucosa, and mild epigastric tenderness. There was no visible swelling around the neck or palpable nodularity of the thyroid gland. The rest of the systemic examination was unremarkable. 

**Figure 1:**
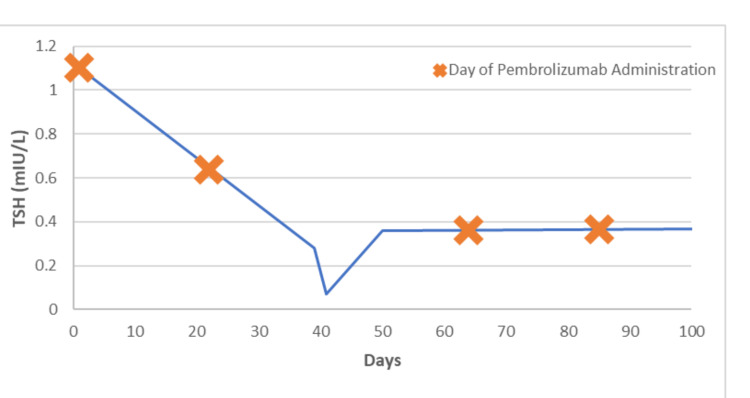
Depiction of TSH levels in relation to pembrolizumab administration TSH: Thyroid stimulating hormone

Pertinent laboratory work on admission included: blood glucose 451 mg/dl, beta-hydroxybutyrate 8.63 mmol/L, glycated hemoglobin (HbA1c) 7.2%, arterial blood gas with pH 7.06, partial pressure of carbon dioxide (pCO2) 17 mmHg, partial pressure of oxygen (pO2) 153 mmHg, bicarbonate (HCO3) 5 mEq/L, lactic acid 2.10 mmol/L, thyroid stimulating hormone (TSH) 0.070mIU/L, free thyroxine 1.85mIU/L. Urinalysis showed +4 glucose and 4+ ketones. Thyroid peroxidase antibodies and TSH binding inhibitory immunoglobulin were within normal limits. TSH was 1.1 mIU/L and blood glucose was 121 mg/dl prior to starting pembrolizumab, which was around six weeks before this admission. Laboratory data of the patient are shown in Table 1. The trend of TSH levels with pembrolizumab administration is depicted in Figure 1. Electrocardiogram (EKG) showed sinus tachycardia with premature ventricular complexes. Thyroid ultrasound showed sub centimeter bilateral thyroid nodules. The chest CT scan did not show evidence of pulmonary embolism. The bilateral lungs were clear without confluent infiltrate, pleural effusion, mass, or pneumothorax.

**Table 1:** Laboratory parameters along with reference range TSH: Thyroid stimulating hormone fT4: Free thyroxine HbA1C: Glycated hemoglobin TPO: Thyroid peroxidase antibodies * Resulted after discharge

Labs	Values	Reference ranges
TSH	0.07	0.270 - 4.20 mIU/L
fT4, ng/dL	1.85	0.93 - 1.70 ng/dL
TPO Antibodies, IU/ml	<0.30	0 - 9.0
TSH Binding Inhibitory Immunoglobulin, IU/L	<0.90	≤1.75
Glutamic Acid Decarboxylase Antibody, IU/mL	<5.0*	0 - 5.0
C-Peptide, ng/ml	<0.1*	0.8 - 3.5
HbA1c, %	7.20%	3.6 - 5.6
Plasma glucose, mg/dL	583	65 - 105
Beta-Hydroxybutyrate, mmol/L	8.63	<0.3
Sodium, mEq/L	131	136-145
Potassium, mEq/L	5.4	3.5 - 5.1
Chloride, mEq/L	99	98 - 108
Bicarbonate, mEq/L	4.8	22 - 26
Anion Gap, mEq/L	27	10 - 18
Blood Urea Nitrogen, mg/dL	25	8 - 21
Creatinine, mg/dL	1.6	0.6 - 1.2
Venous pH	7.06	7.31-7.41
pCO2, mmHg	17	41 - 51
Lactic Acid, mmol/L	2.1	0.5 - 1.6
Prolactin, ng/ml	6.6	2.1 - 17.7
Random cortisol, ug/dL	33	6.2-19.4

The patient was treated with a standardized diabetic ketoacidosis (DKA) protocol that included vigorous intravenous hydration and intravenous insulin. The patient was started on propranolol due to concern for tachycardia and hyperthyroidism, which eventually improved. The DKA resolved and he was transitioned to subcutaneous insulin glargine and insulin aspart. The possibility of hypophysitis was raised during hospitalization due to the co-occurrence of endocrinopathies. A brain magnetic resonance imaging revealed a normal pituitary gland. A random morning cortisol was elevated at 33 ug/dL with normal prolactin level. The patient was discharged with subcutaneous insulin at home. He was advised to follow-up with endocrinology. Anti-glutamic acid decarboxylase (anti-GAD) was negative and TSH normal at follow-up.

At follow-up, PET scan revealed metabolically active lesions in the posterior of the esophagus that were compatible with recurrent esophageal carcinoma with no evidence of distant metastases. Three weeks after discharge, the patient was restarted on pembrolizumab, however, he only received two more doses with a total of four doses of pembrolizumab. After discharge, he had presented to the outside hospital twice for acute food impaction and underwent esophageal stenting. Four months after the first presentation, the patient was readmitted to our hospital with DKA secondary to noncompliance with medications, severe dysphagia, worsening cachexia, and failure to thrive. CT C/A/P showed multiple nodules in the right adrenal gland, lungs, soft tissue of the left flank, and mediastinal lymphadenopathy concerning metastasis. The palliative team was consulted and care was transitioned to home hospice, however, on the day of discharge, he was lethargic, tachypneic and ultimately died of respiratory failure.

## Discussion

The introduction of immunotherapy drugs such as the immune checkpoint inhibitor pembrolizumab has expanded the spectrum of tumors that can be treated, including GE junction adenocarcinoma [6]. The precise mechanism behind the emergence of irAE has not yet been fully understood. One theory of the onset of diabetes is that the absence of inhibitory mechanisms causes autoreactive T lymphocytes to target pancreatic beta cells [7].

In patients treated with pembrolizumab after approval, hyperglycemic events were reported in 45% to 49% of patients with 3% to 6% experiencing grade 3 or 4 hyperglycemic events and fulminant DM1 occurred with an incidence rate of 0.1% [8]. According to a study conducted at the Mayo Clinic, approximately 15% of patients had thyroid dysfunction, which was manifested most frequently as acute, painless thyroiditis or obvious hypothyroidism, and the median time for thyroid problems to appear after starting ipilimumab or pembrolizumab medication was two to four weeks [9]. There is limited data on concurrent endocrinopathies in patients receiving PD-1 inhibitors with only a few case reports of DKA and hypothyroidism after treatment with a PD-1 inhibitor [10,11]. Combined immunotherapy regimens (such as nivolumab/ipilimumab) were more associated with the development of multiple endocrinopathies [12]. In our case, pembrolizumab monotherapy developed two irAE (hyperthyroidism and DKA).

According to a recent meta-analysis, the majority of patients experienced new-onset DM1 in three months, with a median duration of diabetes with PD-1/PD-L1 inhibitors of 49 days (range: 5-448 days) [13]. In the acute care context, it is critical to understand the differences between DM1 and ICI-related diabetes mellitus (DM) in terms of clinical presentation. In 98% of patients with fulminant DM1, increased pancreatic enzymes (amylase and lipase) are observed, although this is uncommon in ICI-related DM [14]. Although less frequent in reports of ICI-related DM, flu-like symptoms were nevertheless frequently observed in fulminant DM1 [15].

Regarding whether islet autoantibodies are associated with ICI-related DM, there is mixed evidence in the literature. Compared to patients with DM1, some studies show that most patients have negative autoantibodies, whereas others show that 30% to 50% of cases have at least one islet autoantibody, most frequently anti-GAD. Additionally, patients with positive anti-GAD exhibited a faster onset of ICI-related DM after starting PD-1 inhibitor therapy [16]. The incidence of immunotherapy-related DKA was found to be 0.7% to 1% [4,7,13]. This suggests that DKA is a relatively rare adverse event among patients receiving pembrolizumab. The literature supports treatment with IV insulin and hydration in patients with DKA and then transitioning to subcutaneous insulin upon discharge [7,13].

One of the most common irAE observed in individuals on PD-1 inhibitors is thyroid dysfunction. In a retrospective cohort analysis, 14% of the participants had thyroid dysfunction, and after stopping the medication, 4% had normal thyroid function [17]. The treatment of acute thyroiditis caused by immune modulators is similar to that for other etiologies (for example, symptomatic therapy with beta-adrenergic blockers, if indicated). Although some patients may recover thyroid function, overt hypothyroidism should only be treated with levothyroxine replacement [9]. Close monitoring with regular symptom evaluation along with testing of TSH and free thyroxine (fT4) either prior to each dose of ICI or at two- to three-week intervals is recommended [18].

Our case is consistent with other reported cases in many aspects, but our patient did not have anti-GAD antibodies at the time of diagnosis. In our case, propranolol was used to treat patient's tachycardia, EKG abnormalities, and an additional side effect of decreasing peripheral conversion of active thyroid hormone.

For pembrolizumab and other ICI used in cancer treatment, the European Society for Medical Oncology (ESMO) guidelines recommend routine blood glucose monitoring to identify hyperglycemia early and prevent DKA. This recommendation must be included in cancer treatment regimens [19]. It is crucial to consider ICI as a potential cause of DKA, especially in individuals with a known cancer diagnosis. Endocrinopathies in particular might begin weeks to months after the first dose of the drug, depending on the irAE. While the critical care setting continues to be the primary location for managing DKA, outpatient management with ongoing insulin therapy is typically required, along with a thorough autoimmune workup [20].

## Conclusions

DKA is a frequent initial presentation for DM1 with pembrolizumab treatment, whose incidence is on an increasing trend. Clinicians should be aware of irAE like DKA and hyperthyroidism. Routine monitoring of symptoms and testing of blood glucose and thyroid function test is recommended. It is necessary to enforce the ESMO recommendations, raise awareness of this potentially serious and fatal side effect of pembrolizumab, and educate patients about the signs and symptoms of diabetes.
